# The effect of mindfulness on decision-making, inhibitory control, and impulsivity of substance use disorder in-treatment patients: A randomized clinical trial

**DOI:** 10.1371/journal.pone.0293502

**Published:** 2023-11-07

**Authors:** Ana Paula Gonçalves Donate, Elizeu Coutinho de Macedo, André Bedendo, Itamar Félix Júnior, Giovanna Gonçalves Gallo, Emérita Sátiro Opaleye, Ana Regina Noto

**Affiliations:** 1 Department of Psychobiology, Núcleo de Pesquisa em Saúde e Uso de Substância, Universidade Federal de São Paulo, SP, São Paulo, Brazil; 2 Laboratório de Neurociência Cognitiva e Social, Centro de Ciências Biológicas e da Saúde, Universidade Presbiteriana Mackenzie, SP, São Paulo, Brazil; University of Huelva: Universidad de Huelva, SPAIN

## Abstract

This study aimed to investigate the effects of Mindfulness-Based Relapse Prevention (MBRP) in decision-making, inhibitory control and impulsivity compared to Treatment as Usual (TAU) for individuals with Substance Use Disorders (SUD’s) in Brazil. A randomized clinical trial was conducted with participants from a therapeutic community (n = 122). Decision-making (Iowa Gambling Task), impulsivity dimensions (UPPS-P Scale), and inhibitory control (Stroop Color-Word Test) were assessed before and after the MBRP 8-week intervention. GLM Multivariate analysis was used to evaluate the effects of MBRP on different impulsivity measures. The results showed that MBRP+TAU improved the general decision-making score (p = 0,008) compared to TAU. However, no significant effects were found in impulsivity dimensions and inhibitory control in individuals with SUDs in the therapeutic community. This study found improvement in decision-making in the total IGT score; however, no effect for self-reported impulsivity and inhibitory control among middle-aged patients after an 8-weeks intervention of MBRP protocol in an inpatient setting. It adds information to the subject, with implications and possible directions to be followed by the next clinical trials with patients with SUDs in treatment.

**Trial registration**: EnsaiosClinicos.gov.br: RBR-6c9njc.

## Introduction

Around 35.6 million people between the ages of 15–64 suffer from substance use disorders (SUD) worldwide, corresponding to 0.7% of the global population [1]. In Brazil, 1.6 million have received treatment for SUDs, and one of the most common treatment settings is Therapeutic Community, where patients with SUDs are referred to receive treatment as usual [2]. Treatment as usual (TAU) is the standard treatment offered in a clinical condition and can be combined with mindfulness meditation [3].

Besides the workforce developing a treatment for SUDs, offering effective treatments for this population remains challenging due to their harmful consumption, relapse rates, and low treatment retention. Previous evidence also suggested that impulsivity contributes to substance use. Likewise, drug use also increases impulsivity levels and contributes to the intensification of the behavior by impairing inhibitory control and decision-making. Therefore, even in the face of the severe negative consequences of its use, it is challenging to prevent relapse [4].

Impulsivity is a multidimensional construct often referred to as a tendency to act quickly and react to external or internal stimuli without reflecting upon the consequences [5]. The most contemporary theory divides the construct into five distinct domains, which are: negative urgency, referring to the tendency to act precipitously and having strong impulses when experiencing negative emotions, usually accompanied by regret after acting; lack of premeditation, reflecting the tendency of not thinking about consequences or planning before acting; lack of perseverance, which refers to the lack of ability to focus and accomplish activities; sensation seeking, which is the tendency to seek adventure, enthusiasm and exciting activities that may put one’s life at risk; and positive urgency, related to the tendency of acting rashly when experiencing positive emotions [6].

Furthermore, impulsivity has two main mechanisms: inhibitory control, which is the capacity to stop an automatized behavior that has already started, and decision-making, which is the ability to decide from the consequences and evaluate long-term benefits over short-term gains [7]. Impulsivity can be measured both through self-report questionnaires [8] and through laboratory-based tasks in behavior psychology [5, 6, 9, 10], which are based, respectively, on the mentioned theories.

According to the Dual-System Theory, our cognitive system is divided into automatic and controlled processes [11]. The automatic system is impulsive, depending on previous learning processes, while the controlled system is slow and requires evaluation for further decision-making. In SUDs, the impulsive system relies on automatized substance use behavior despite negative consequences. Substance use can be related to bottom-up mechanisms being suppressed by automatic or reward-driven responses due the diminished cognitive control [11]. The imbalance between two systems: a top-down (controlled), goal-oriented and reflective (frontostriatal circuitry dependent), and a bottom-up (automatic), characterized by a quick and without reflection answer based on habitual response (amygdala dependent). Mindfulness seems to operate into affective and cognitive mechanisms involved in impulsivity, inhibitory control, and decision-making [12].

Mindfulness may refer to both a psychological state and a meditation practice. The practice intends to promote the state of mind of being aware of the present moment or experience without reacting to, avoiding, or judging it (Medical Subject Headings, MeSH Unique ID: D064866). Mindfulness-Based Relapse Prevention (MBRP) is a specific 8-weeks (2h/week) protocol that addresses addiction and reactive behaviors, and includes relapse prevention approaches and meditation practices. MBRP is a promising complementary intervention and posttreatment for SUDs [13]. The protocol focuses on developing the autoregulation of the attention with awareness for the present moment experiences (both internal, such as emotions and sensations, and external, such as songs or temperature) without judgment or reaction [14, 15]. It aims at increasing awareness with acceptance of the present moment without reacting automatically.

Previously cross-sectional analysis pointed out that different aspects of impulsivity are inversely related to specific dimensions of mindfulness in people with alcohol use disorder in therapeutic community treatment, meaning that higher mindfulness traits were associated with less impulsive traits [16]. Also, lower rates of substance use by patients have been observed during and after the MBRP intervention [13, 17], as well as less severe substance use disorders were observed compared to TAU [18]. Moreover, studies have shown that MBRP participants may present a reduction of craving immediately after the treatment and in the long-term [13], and that there are changes in impulsivity domains in a young population with SUDs after an MBRP twice-a-week intervention compared to a 12 steps/self-help protocol [19]. When it comes to populations with specific substance use disorders, previous studies have shown decreased level of urgency in a small sample of patients with alcohol-use disorders, as well as a reduction of the general level of impulsivity in patients treated with methadone after the intervention and during the follow-up in the MBRP group when compared to TAU [20, 21].

The vast amount of literature on the impact of SUDs worldwide highlights the importance of treatments capable of reducing harmful drug use. In this specific treatment environment, we cannot use substance use level as an outcome because the patients do not have access to substances. Then, as impulsivity is risky for relapse and understanding mindfulness mechanisms over impulsivity, the MBRP seems to be a treatment possibility. Second, an investigation on impulsivity using only a questionnaire could not capture the nuances of this complex cognitive process, demanding a combination of techniques such as behavioral tests. Innovative treatments designed to improve impulsivity outcomes such as reactivity would not only reduce the risk of relapse for people with SUD likewise to offer improvement in other aspects of their lives, such as general well-being and interpersonal relationships.

Therefore, this study was developed to investigate the effects of an 8-week MBRP program on impulsivity during treatment in an inpatient setting in Brazil. To the best of our knowledge, this is the first study to do so in a therapeutic community through impulsivity self-report questionnaires and behavioral tests evaluating an 8-week MBRP intervention [3]. We hypothesized that MBRP+TAU would lead to lower rates of impulsivity in self-report questionnaires, better decision-making rates, and more inhibitory control compared to TAU at post-intervention.

## Methods

### Study design

This is a randomized clinical trial study, non-blinded, controlled, 1:1 with pre-post intervention comparison.

**Fig 1:**
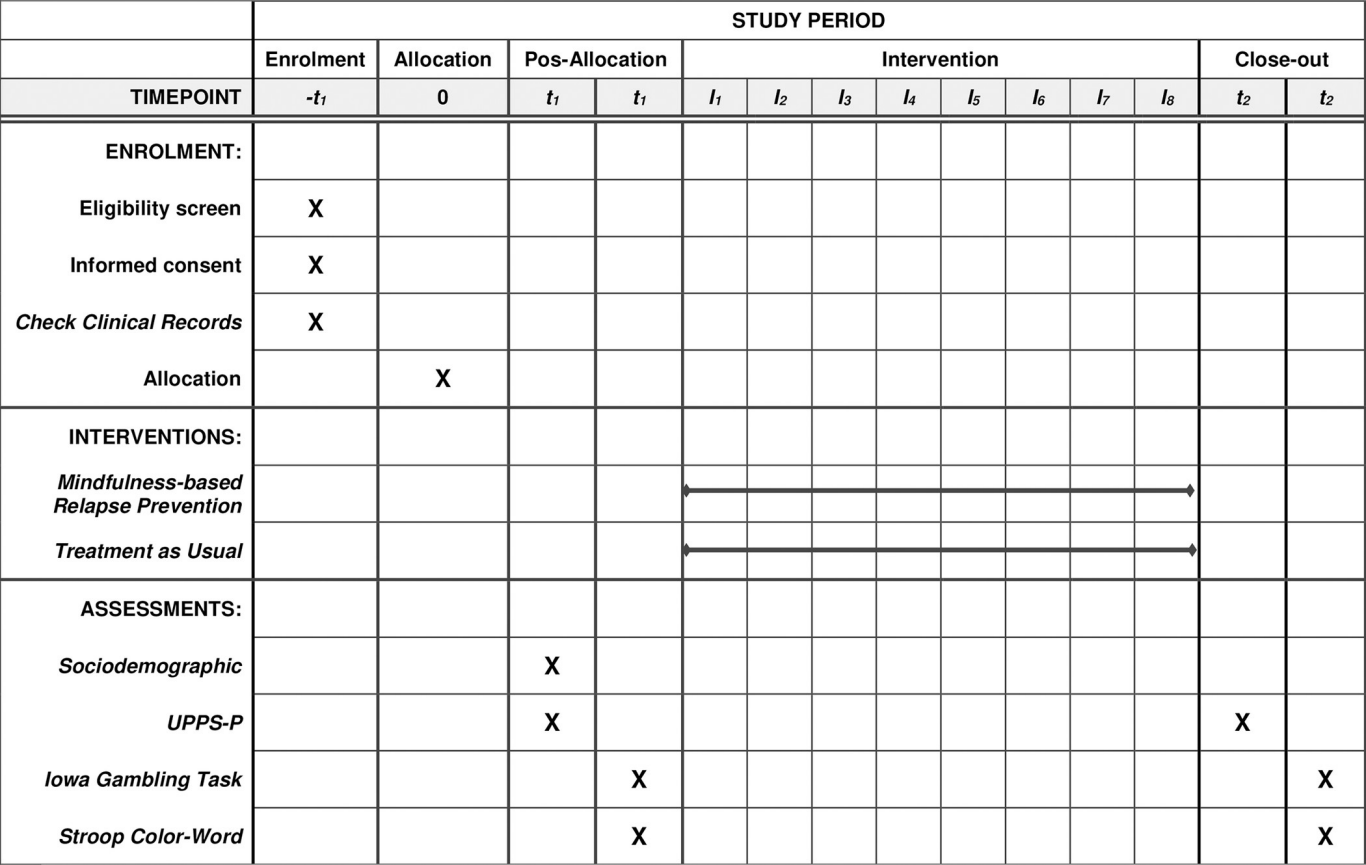
SPIRIT schedule presenting participant timeline.

This article was written according to SPIRIT Guidelines [22] and CONSORT-PSI [23]. All participants were randomized between TAU or MBRP+TAU groups. Outcome measures were assessed two times: at pre-intervention (2 weeks before starting the intervention) and at post-intervention (1 or 2 weeks after the 8-week intervention). Fig 1 shows in detail the instrument that was applied and the times of their administration.

### Study setting

The participants were in a closed treatment for SUDs in a Therapeutic Community (TC), where people with SUDs stayed voluntarily or involuntarily to recover from the disorder in a controlled environment during a limited period [3]. This treatment facility receives people living in conditions of homelessness, any other social vulnerability. To start the treatment at the institution, the patient had to be without substance use for at least a day. The facility had a capacity of 100 people who could live there for a maximum of 6 months. Those who met the inclusion and exclusion criteria and agreed to participate were randomized between two groups: MBRP + TAU or TAU after answering the self-report measures and behavioral tasks. In addition, each TC had its own team of psychologists, social workers, advisors, and a psychiatrist.

### Eligibility criteria

The Therapeutic Community is restricted to individuals with a substance use disorder referred to inpatient treatment by social services, health services or even by themselves. This study has followed the Helsinki Declaration. The participants were informed about the purpose of the study, potential benefits and risks, and their right to refuse participation or withdraw without penalties. All participants and the staff were informed and clarified about the inclusion and exclusion criteria. All participants provided their written consent.

The study was submitted, approved, and conducted based on the requirements from the Research Ethical Committee (CEP) of the Federal University of São Paulo (#2.756.612 with amendments: #2.782.832 and #3.557.636). It was also registered and approved by the Brazilian Clinical Trials Registry (EnsaiosClinicos.gov.br: RBR-6c9njc).

**Fig 2:**
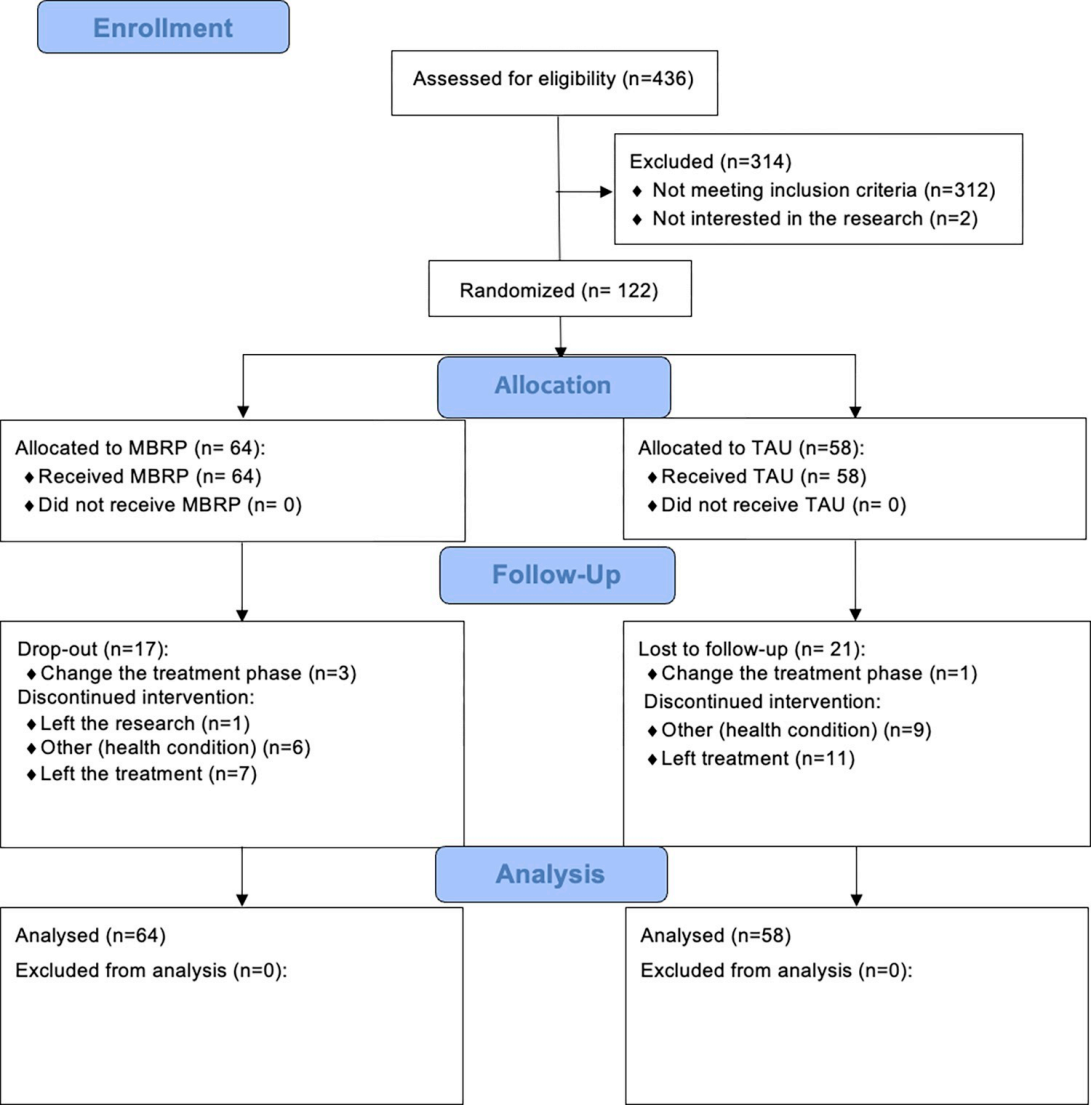
CONSORT flow diagram.

Randomized participants for the control group were offered the opportunity to receive the MBRP protocol after the therapeutic community treatment ended. They did not receive any compensation for their participation. Fig 2 shows a flow diagram of the study design of the MBRP and TAU based on the CONSORT-PSI [23] Diagram.

The inclusion criteria were that participants must: 1. Be 18 years old or older; 2. Have Portuguese as their first language; 3. Were receiving at the study period an inpatient treatment for substance use disorder of any kind; 4. Had been in the service for at least 15 days and therefore, reporting no drug consumption for this period (except coffee or tobacco); 5. Accept to participate in the research. Their report on substance abstinence was double checked with the institution staff and also via their medical record. Besides, participants do not leave the institution without permission, and anyone inside the institution is allowed to have any psychoactive substance, except for caffeine and tobacco.

The exclusion criteria were: 1. Were diagnosed with severe neurological or psychiatric diseases; 2. Had suicidal ideation or posed a risk to themselves and others; 3. Consumption of substance within the last 15 days; 4. Had psychotic disorders. All the information was cross-checked with the patient’s medical records from the institution and the therapeutic community team.

### Interventions

#### MBRP+TAU

The MBRP+TAU group was structured to happen in eight weekly meetings of two hours each. Therapists delivered MBRP to groups of 5–15 participants in each cohort. The protocol aims at becoming aware of triggers, habitual patterns, and automatic reactions that permeate the addictive process [13]. During these meetings, four main meditation techniques were practiced: breathing, body scanning, meditative walking, and mindfulness movements. The specific themes of each session were: 1 –Automatic Pilot and relapse; 2 –Awareness of triggers and craving; 3—Mindfulness in daily life; 4—Mindfulness in high-risk situations; 5—Acceptance and skillful action; 6—Seeing thoughts as thoughts; 7—Self-care and lifestyle balance; 8—Social support and continuing practice. At the end of each session, a guide was given with instructions to MBRP participants containing basic concepts discussed during the session and orientation to practice meditation during the week. The intervention groups were conducted by instructors holding a psychology Bachelor (4), background or specialization in cognitive-behavioral therapy (2), and doctoral degree (1) with meditative practice and habilitation in the referred program, trained and supervised at Medita NEPSIS (national reference center in Brazil). All sessions were audio and written recorded during the research. Treatment fidelity was assessed to identify key content- and style-related components of MBRP, using the number of practices delivered as a reference.

#### TAU

The TAU received the usual treatment delivered by the TC staff. It was structured and planned by the institution, follows the determinations of the Brazilian Federation of Therapeutic Communities (FEBRACT), and includes spirituality activities, 12 steps, relapse prevention, Singular Care Plan, and physical education, divided into phases (welcoming, treatment and social reintegration). All participants (both in TAU and MBRP+TAU) were in an educational process based on mutual-help principles, relapse prevention, and 12-step intervention [24].

### MBRP fidelity

MBRP sessions were recorded with a recorder without internet access and were evaluated by an independent reviser. We used an Excel sheet graded from 0 to 67 to evaluate the fidelity, which is the total number of protocol activities. The group activities were graded by the number of activities divided by the number of completed activities. The mean of group activities was 95% (between 92% to 100%). The mean attendance rate was 77%.

### Sample size

The sample size was calculated using G*Power Software version 3.1.9.4 considering a significance level of 5%, 80% observed power and 5% of effect size on the primary outcome (impulsivity) and secondary outcome (inhibitory control and decision making) for the intervention. Calculations were based on the primary outcome. Using these criteria, we needed 101 participants (MBRP+TAU = 51; TAU = 51). The allocation ratio of 1:1.

### Recruitment

The recruitment process happened from July 2018 to December 2019. This research was conducted in an inpatient treatment facility called therapeutic community located in Sao Paulo—Brazil that exclusively offers treatment for substance use disorders. Recruitment for study happened when the patients were already interned receiving treatment for substance use disorders in the institution for at least 15 days through mechanisms such as advertising the research in a small meeting organized by the staff to invite potential participants.

### Measures

Research assistants administered the questionnaires and the behavioral tests after receiving training from APGD. The participants answered it at the pre-intervention (two weeks before the intervention) and post-intervention (during the next two weeks, followed by the end of the intervention). Each approach was conducted on different days and weeks, and the sequence of the measures was:

#### Self-report measures

Sociodemographic data questionnaire: age, gender, monthly income, educational level, substance use, relationship status, and previous treatments for drug use.

UPPS-P: A 20-item questionnaire assessing the impulsivity facets: Perseverance, Premeditation, Sensation Seeking, Positive Urgency, and Negative Urgency. Each item is measured using a 1–4 Likert scale, and the questionnaire has already been validated in Brazil [6, 25]. With the presentation of the subscales means, meaning that higher scores correspond to higher impulsivity in each dimension [25]. All subscales demonstrated acceptable or good internal consistency. Cronbach’s alpha coefficient: total UPPS-P (α = 0.75); Perseverance (α = 0.59); Premeditation (α = 0.73); Sensation Seeking (α = 0.59); Positive Urgency (α = 0.68); Negative Urgency (α = 0.64).

#### Behavioral tasks

Stroop Word Color Test: The test evaluates the inhibitory control ability ([26]). The version used had three cards to which participants were asked to respond as quickly as possible, from left to right. For the first card, participants were asked to say the colors in which the words were written on the card; for the second card, they were asked to read the word written on the card; and for the last card, participants were asked to say what color that a word was written in, with the word being a word for a color that did not match the actual color used on the card. We used the Stroop Effect of time and error to score the test and the calculated z-score.

Iowa Gambling Test (versions: A’B’C’D’ and K’L’M’N’): The computerized task which evaluates learning and decision-making in reward and punishment situations in uncertain conditions [27, 28]. During the test, participants must choose cards one hundred times from a choice of four different decks. Each card chosen results in a monetary gain or loss for the participant. Each deck is weighed towards being either positive or negative–decks A and B are disadvantageous, and decks C and D are advantageous. Positive cards from decks A and B simulate reward participants with R$100 to R$250, while decks C and D only reward R$50. However, decks A and B also have larger losses than decks C and D; overall, decks C and D are more profitable for the participants. The formula (A+B)-(C+D) calculates the Total-SCORE. Positive scores mean that participants perform well and learn from the consequences. Then post-intervention evaluation, a different version of IGT was applied based on K’L’M’N’ to prevent the learning effect from the previous version [29].

### Procedure

**Fig 3:**
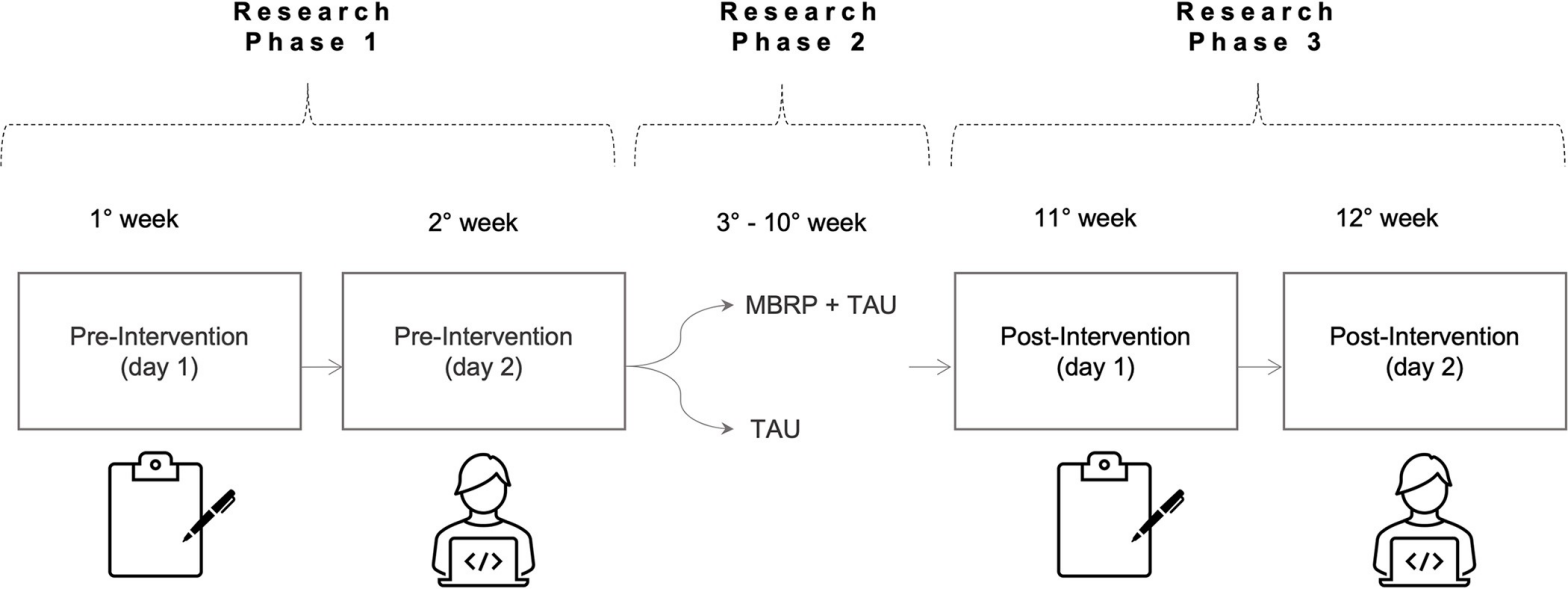
Fluxogram of the experimental design.

The protocol had three phases: 1. Pre-intervention; 2. The intervention delivered to participants in treatment; and 3. Post-intervention, as represented in Fig 3. All phases happened on consecutive Mondays, and four separate rooms were available for the research, three of which were used for data collection of each behavioral test and the fourth one for the delivery of the MBRP protocol.

### Allocation

The randomization of the participants was conducted by the site (https//:www.random.com) using a code number for each participant, by aleatory number generation, and under the supervision of other researchers with no relationship with the current research. The participants were assigned to either MBRP+TAU or TAU with a 1:1 allocation. The research assistants responsible for collecting data were blind to the participants’ groups.

### Data management

#### Data management

Data collection used paper-and-pen self-reported measures and a computer for behavioral tasks. All the participant forms were stored in numerical order and in a secure place at the Research Center for Health and Substance Use (NEPSIS). The electronic data from the behavioral tasks are stored in a computer with a password to access. Additionally, all data was added to REDCap, and a double conference was conducted to run the analysis.

#### Data analysis

Analyzes used Generalized Linear Models (GLM) to assess the effects of time, group and, the intervention over time compared with the control group for the following outcomes (dependent variables): a) UPPS-P: Perseverance, Premeditation, Sensation Seeking, Positive Urgency, Negative Urgency, Emotion-based Rash Action (formed by Negative and Positive Urgency) and, Deficits in Conscientiousness (formed by lack of Perseverance and Premeditation); b) IGT A’B’C’D’ = Total NET-Score [disadvantage beck: (A+B)]—[advantage deck: (C+D)] and each block, IGT K’L’M’N’ = Total NET-Score [disadvantage beck: (M+N)]—[advantage deck: (K+L)] and each block c) Stroop Word-Color Test: Stroop Effect for Time and Number of Errors. All the outcomes’ scores were analyzed with a 2 (time: pre, post-intervention) × 2 (condition: MBRP+TAU, TAU) multivariate GLM. The models considered Gaussian Distribution, and the best distribution was chosen based on the smallest Akaike Information Criterion (AIC) values. All models were adjusted for pre-intervention, age, and gender. All participants data was included in analyzed. A Bonferroni correction for multiple comparisons was made to account for having three outcomes, independently of being primary or secondary, and the *P* value for statistical significance was p = 0.0167. For missing data, we used pairwise deletion, using all information provided by participants to avoid the bias caused by listwise deletion. The analyses were conducted in the R Studio Software [30] 4.0.2 version.

## Results

### Demographic characteristics

A total of 122 participants consented to participate in the randomized controlled trial. All participants answered at least one of the principal outcomes. Most of the sample responded self-report and behavioral measures (n = 86) attrition rate 32%, and responded only self-report (n = 36) attrition rate 28%. Any participant reported harm or side effects.

Descriptive information about the participants’ characteristics is shown in Table 1. Most participants were male [n = 111 (91%); for MBRP*time MBRP+TAU: n = 64; TAU: n = 58] medium age of 41 years (±10.3) and mostly single (n = 62; 49.2%) or divorced/ widower (n = 42; 35%). Most participants had at least complete/ incomplete middle or high school (n = 54; 45%) and had no monthly income (n = 26; 31%), which wages were R$954,00 in 2018 (price of Dollar in 2018: $1 = R$3.88) (n = 26; 31%)]. The average days of abstinence was 66.2 days between participants at the pre-intervention. Randomization was successful, and groups were similar at the pre-intervention.

**Table 1:** Sociodemographic and substance use information of participants at baseline.

Baseline Characteristics	MBRP		TAU		Full sample	
	*n*	%	*n*	%	*n*	%
Participants	64	-	58	-	122	-
Age (mean)	64	42.1	58	39.7	122	41
Gender						
Male	58	90.6	53	91	111	91
Female	6	9.4	5	8.6	11	9
Monthly Income						
Without income	11	25.6	15	36.6	26	31
1–2 minimum wages*	4	9.3	3	7.3	7	8.3
3 minimum wages	16	37.2	9	22	25	29.8
4–5 minimum wages	7	16.3	8	19.5	15	17.9
6+ minimum wages	5	11.6	6	14.6	11	13.1
Marital Status						
Single	27	42.9	32	56.1	59	49.2
Married	12	19	7	12.3	19	15.8
Widow or divorced	24	38.1	18	31.6	42	35
Education**						
Elementary School	20	31.2	20	35.7	40	33.3
Middle or High School	28	43.8	26	46.4	54	45
Higher Education	16	25	10	17.9	26	21.7
Age of First Treatment (mean)	52	31.2	50	30.8	102	31
How many times have you engaged in treatment?						
Two or less	41	35	39	33.3	80	68.4
Three or more	20	17.1	17	14.5	37	31.6
Substance Reported as Currently More Challenging						
Alcohol	32	54.2	30	65.2	62	59
Cocaine / Crack/ Merla	21	35.6	12	26	34	31.5
Marijuana	0	0	2	4.3	2	1.9
Tobacco	6	10.2	2	4.3	8	7.6
Days in Abstinence (mean)	63	65.3	57	67	120	66.2

Note. *N* = 86 (MBRP = 64; TAU = 58). Participants were on average 41 years old (*SD* = 10.3), and participant age did not differ by condition (p = .185).

** Reflects complete or incomplete education level.

*P* < = 0.05.

Information about the participants’ descriptive responses to questionnaires and behavioral tests is shown in Table 2.

**Table 2:** Participants’ descriptive responses to questionnaires and behavioral tests at baseline.

	Pre-intervention		Post-intervention	
	MBRP+TAU	TAU	MBRP+TAU	TAU
	*μ (SD)*	*μ (SD)*	*μ (SD)*	*μ (SD)*
UPPS-P				
Lack of Perseverance	7.0 (2.3)	7.2 (2.7)	6.4 (2.3)	6.2 (1.5)
Lack of Premeditation	7.9 (2.8)	7.9 (2.9)	7.0 (2.5)	7.0 (1.6)
Negative Urgency	10.5 (3.3)	10.4 (3.0)	9.9 (2.7)	10.2 (3.1)
Positive Urgency	10.0 (2.9)	9.7 (3.0)	9.8 (2.7)	9.9 (3.1)
Sensation Seeking	10.4 (3.6)	10.3 (3.0)	10.6 (2.8)	10.1 (3.5)
Emotion Based Rash Action	20.5 (5.7)	20.2 (5.3)	19.8 (4.8)	20.1 (5.7)
Deficit Conscientious	14.9 (4.5)	15.1 (5.2)	13.4 (4.4)	13.1 (2.6)
Iowa Gambling Task				
Total Score	-2.7 (17.1)	-9.2 (19.0)	4.5 (27.6)	-14.1 (21.9)
Block 1	-2.4 (5.0)	-1.8 (4.5)	0.6 (7.3)	-1.8 (6.9)
Block 2	-0.5 (6.0)	-0.9 (4.9)	-0.6 (7.8)	-0.9 (7.7)
Block 3	-1.5 (6.7)	-1.7 (6.9)	2.0 (9.0)	-1.6 (7.2)
Block 4	-0.3 (8.0)	-3.8 (6.7)	0.4 (10.5)	-6.4 (7.2)
Block 5	1.6 (7.2)	-1.1 (6.8)	1.2 (8.3)	-0.6 (10.5)
Stroop Word Color Test				
Stroop Effect	-0.1 (0.8)	0.1 (1.2)	-0.0 (0.9)	0.1 (1.1)
Stroop Effect (error)	0.1 (1.1)	-0.1 (0.9)	-0.0 (1.0)	0.1 (1.0)

*Note*: All the outcomes were evaluated and we used average (*μ)* and standard deviation (SD). For multiple comparisons, we applied Bonferroni correction.

### IGT

IGT results are shown in Table 3. There were no significant differences for time and intervention, but a significant effect for the interaction MBRP*time = 14.12, 3.79–24.5, *p* = 0.008 was found for IGT Total score. Regarding the specific blocks, Block 1 was statistically significant for interaction for MBRP*time = 3.87, 0.39–7.34, *p* = 0.03. The effect size for the IGT Total Score was considered medium (*d* = 0.733) and for IGT Block 1, small (*d* = 0.337), Block 2 (*d* = 0.028), Block 3 (*d* = 0.430), Block 4 (*d* = 0.742), Block 5 (*d* = 0.189). However, we did not find effects on the specific blocks after Bonferroni corrections were applied.

**Table 3:** GLM of Iowa gambling test (IGT), self-report impulsivity (UPPS-P) and stroop color task.

	Predictors	Estimate	95% CI		*p*	Estimate	95% CI		*p*	Estimate	95% CI		*p*	Estimate	95% CI		*p*	Estimate	95% CI		*p*	Estimate	95% CI		*p*
IGT		IGT Total Score				Block 1				Block 2				Block 3				Block 4				Block 5			
	Group ^MBRP^	1,98	-4,64	8,59	0,55	-0,04	-2,25	2,16	0,96	0,32	-1,9	2,6	0,8	0,15	-2,1	2,4	0,9	1,35	-1,8	4,5	0,39	1,44	-2,02	4,89	0,41
	Time	-6,02	-13,94	1,89	0,13	-0,78	-3,44	1,89	0,56	-1,03	-3,8	1,7	0,5	0,35	-2,4	3	0,8	-3,06	-6,8	0,6	0,1	1,51	-2,64	5,66	0,47
	MBRP*Time	14,12	3,79	24,5	**0,008**	3,87	0,39	7,34	0,03	1,55	-2	5,1	0,4	3,36	-0,2	6,9	0,1	3,74	-1,1	8,6	0,12	-2,16	-7,57	3,25	0,43
UPPS-P		Lack of Premeditation				Negative Urgency				Positive Urgency				Sensation Seeking				Lack of Perseverance							
	Group ^MBRP^	-0,01	-0,56	0,55	0,97	0,05	-0,7	0,8	0,89	0,12	-0,6	0,8	0,7	0	-0,6	0,6	1	-0,07	-0,6	0,5	0,81				
	Time	-0,45	-1,09	0,19	0,16	-0,23	-1,09	0,64	0,6	-0,1	-0,9	0,7	0,8	-0,19	-0,9	0,5	0,6	-0,93	-1,6	-0,3	0,01				
	MBRP*Time	-0,51	-1,38	0,36	0,25	-0,38	-1,56	0,8	0,52	0,06	-1	1,1	0,9	0,53	-0,4	1,4	0,2	0,37	-0,5	1,3	0,41				
UPPS-P		Deficit Conscientiousness				Emotion Based-Action																			
	Group ^MBRP^	-0,08	-1,06	0,9	0,87	0,17	-1,05	1,39	0,78																
	Time	-1,39	-2,52	-0,26	0,01	-0,33	-1,73	1,08	0,64																
	MBRP*Time	-0,11	-1,65	1,43	0,88	-0,29	-2,21	1,63	0,76																
STROOP		Stroop Effect (time)				Stroop Effect (error)																			
	Group ^MBRP^	-0,04	-0,32	0,24	0,79	0,04	-0,26	0,33	0,81																
	Time	0,11	-0,22	0,45	0,5	0,27	-0,08	0,62	0,12																
	MBRP*Time	-0,2	-0,65	0,24	0,36	-0,34	-0,8	0,12	0,14																

Note: All the outcomes were adjusted for age, gender and baseline. Bonferroni p = 0.0167.

We performed a sensitivity analysis by adding abstinence days as a covariate to the IGT Total score model, which confirmed the significant effect for the interaction MBRP*time = 11.02, 0.67–21.37 p = 0.037.

### UPPS-P

UPPS-P results are shown in Table 3. No significant effects were found for time, intervention, or interaction between the time and the intervention for subscales: Premeditation, Perseverance, Sensation Seeking, Positive Urgency, and Negative Urgency. Concerning the deficit of Conscientiousness (formed by Perseverance and Premeditation), the data analysis showed an effect for Time b = –1.39–2.52 –-0.26, p = 0.01 and the effect size was considered negligible (*d* = 0.0072). However, after Bonferroni’s correction, the result was not confirmed.

### Stroop test

Stroop results are shown in Table 3. There were no effects of group, time, or interaction of MBRP*time.

## Discussion

The literature on impulsivity has already pointed out the challenge of measuring and understanding this construct, which leads to difficulty in intervening on it. With the benefit of investigating different dimensions of impulsivity through multiple measures, this study found the effects of the MBRP group overtime on the total score of the IGT, even though it has not found any improvement in other impulsivity aspects evaluated. Therefore, the hypothesis previously made was partially corroborated and needed to be taken with caution.

The IGT is an instrument for investigating decision options in ambiguous outcomes [31] in which greater performance could be interpreted by better implicit learning from feedback on this task [32]. This study found out that MBRP showed better performance on IGT compared to control groups. However, we cannot affirm that improvement on IGT implies that participants would improve their implicit learning from feedback in daily life. Further studies should investigate the daily life changes in decision-making after mindfulness meditation in this population.

According to the Somatic Marker Hypothesis, people with SUDs have an impaired reasoning and decision-making that contributes to failure in learning with ambiguous or negative consequences of their behavior focusing on immediate reward. Particularly, there is an imbalance between impulsive and reflective systems due to biased interoceptive signals systems promoted and maintained by continuous substance use. Considering this, the treatments for SUDs have been focused on improving the reasoning and decision-making by evaluating and avoiding the activation of those triggers that led to relapse to override the impulsive behavior. However, the relapse rates are still high with these treatment strategies [32, 33].

In our study, we hypothesized that mindfulness training would increase self-awareness and reduce the impulsivity via meditation practices and attitudes taught during the MBRP protocol (e.g., non-judgment, patience, acceptance, letting go, trust, non-striving and beginner’s mind) [12, 18]. Consequently, by these mechanisms, people with SUDs would notice high arousal interoceptive signals without the automatic reaction [34]. Therefore, MBRP training offers a different treatment direction from the traditional treatments for SUDs, which is decoupling the relationship between the trigger and the biased automatic response with mindfulness attitudes learned in the program, instead of avoiding the interoceptive signals from triggers. This would be possible by changing the relationship with the interoceptive signals. In this sense, our findings raise further questions, such as whether it would be beneficial for specific types of substance use disorder, such as cocaine, that should be investigated in future studies.

It was hypothesized that the MBRP+TAU group would have lower rates of impulsivity on specific dimensions over time based on previous literature that observed it in RCTs with patients with SUDs in treatment [19, 21]. However, the present study did not find any significant reduction in impulsivity facets evaluated through the UPPS-P questionnaire when compared to TAU. There are two main possible explanations for these results.

First, the compared studies have essential experimental design differences. For example, a study applied a twice-a-week rolling MBRP protocol with daily supervised thirty minutes practice and compared it to the usual treatment [19]. It has already been pointed out previously by Ross and colleagues the importance of dose/frequency, type of practice and a minimum number of sessions for predicting the decrease of craving and improvement of mental health. It is possible that statistically significant differences in the 8-week MBRP group could be observed with more frequent practices between sessions [35]. Second, previous studies had a homogeneity of substance use [21]. In our study, our sample was composed of people with different SUDs; therefore, it would be possible that we had a range of different impulsivity profiles. There is evidence that different UPPS-P dimensions are related to different SUDs. For instance, negative urgency and lack of premeditation were already related to problematic alcohol use [36], and negative urgency was linked with cocaine use disorder [37]. The variability of levels of impulsivity could contribute to different outcomes in treatment since patients with higher negative urgency, and lack of premeditation have worse treatment outcomes than those who do not [38].

Finally, it is important to consider that there are other main differences in the sample of previous studies, such as age average. Previous RCTs evaluated MBRP in younger patients when compared to our sample [19, 21, 39]. There may be a scientific literature bias of impulsive behavior and addiction, focusing research on adolescence and younger adults because of the critical period of development of SUDs [8, 40]. In our sample, the data collected are from people with a mean of 40.8±10.2 years old.

It is worth observing that middle-aged people could have more severe substance use, neuroplasticity may be more compromised, and they may present more related problems as a consequence of drug use than younger people. Therefore, it seems challenging to understand what the actual cause of the observed impulsivity is, given that it could be a personality trait prior to substance use, or a consequence of it, if the use of the substance specifically contributed to accelerating the expected cognitive decline of aging and, consequently, intervene in those deficits [8, 41]. The fact is that since these cognitive functions are impaired, either by one factor or another, our sample would probably benefit from longer meditation protocols and monitored daily practices, as other studies have offered [8, 19].

### Strengths

The use of multifaceted instruments in the study favored our understanding of this phenomenon involving dependence and impulsivity. In addition, it allowed us to make specific inferences regarding cortical functioning from the results obtained by scales and behavioral tests. Also, it is noteworthy that it was a controlled and randomized experimental design, which contributed to testing causal hypotheses about possible impacts in impulsivity after an eight-week MBRP protocol.

Another strength of our work was that it was conducted in a therapeutic community, which offers a structured place and stable routine with basic resources for recovery, while still offering the challenges inherent to substance use disorders treatment environments.

In addition, this study fills an important gap in the science of psychology whose results have relied on sampling WEIRD (Western, educated, industrialized, rich, and democratic) populations. Also, the fact the sample of middle-aged patients being treated for various substances is also innovative, considering that studies with SUDs usually have a bias towards adolescents or young adult patients, as they are considered a population at high risk. Consequently, it increases the difficulty in better understanding and promoting effective interventions, such as MBRP, for middle-aged adults with SUDs [8].

### Limitations and future directions

Despite the positive sides, the highly controlled environment, a characteristic of the Therapeutic Community, is also a limitation of the study since the attended patients used to live alone or as homeless people, facing violence and financial/food deprivation daily. This fact has two possible important implications: patients were not exposed to triggering circumstances, unlike previous studies that reported effects on impulsivity in different settings [42]; and, for being there, patients had a different lifestyle than they used to, and had no access to the “real world” and usual context of the use of substances.

The main limitation in consequence of the chosen setting is that a clinical trial running inside a closed environment has a higher contamination risk. In an attempt to minimize this bias, we establish a confidentiality agreement before the beginning of the intervention. Also, participants were not allowed by the service to receive devices to use audio, eliminating the chance of exchanging recorded meditation inside the community. Howsoever, we have no guarantee that the MBRP participants did not communicate with control ones about the intervention concepts since they were in daily, close contact.

On the other hand, another limitation was the lack of access to the audio for the meditation practices throughout the week. They received a booklet with the basic concepts and names of the practices and were asked to practice daily. The use of guided meditation is a way to facilitate the practice outside sessions and can be a strategy for learning the myriad of meditations taught in the program. One has to consider that harm or side effects interfere in their adherence to practice and we did not actively question it.

A third limitation, the periodic external appointments (justice, social assistance, or medical), prevented some participants from attending all sessions while others were discharged, which eventually impacted our dropout rate. Besides this, we did not evaluate the comorbidities of these patients. It could represent a possible direction for future studies, since this group seems to have an impairment in executive functions both because of substance use itself and because of comorbidities [43].

Finally, the lack of information on years of substance use or severity of dependence can constitute a limitation as this may impact their impulsivity profile. one may consider the age range of our sample as a limitation that reduces comparability to most of RCTs on mindfulness, impulsivity, and addiction, carried out predominantly among younger adults. Future possible directions for studies are to investigate the effects of MBRP on impulsivity dimensions [44] and behavioral tasks in a rolling MBRP group with a larger middle age sample with specific SUDs and monitored practices between sessions, also increasing the follow-up time.

Considering the findings of our study, future perspectives emerge about the importance of a greater number of meditation sessions in this setting and age group while considering a multifaceted approach to impulsiveness. It is believed that the number of sessions and hours of meditation can improve impulsivity since patients who were offered more than 12 [8] and 8 with monitored practice between sessions improved their impulsiveness[40]. Another direction that could increase our understanding on the effects of mindfulness on impulsivity would be to apply substance-related impulsivity instruments, such Stroop Alcohol test, which unfortunately was not possible in our study due the variability of substance consumption in our sample [45]. Furthermore, it would be interesting to have studies of mediators and moderators on the effect of the MBRP intervention on impulsiveness to improve the replicability [46].

## Conclusion

This study contributed for improvement in executive functions, specifically decision-making and no effect for self-reported impulsivity and inhibitory control among middle-aged patients after an 8-weeks intervention of MBRP protocol in an inpatient setting. It adds information to the subject, with implications and possible directions to be followed by the next clinical trials and basic psychology research with patients with SUDs in treatment.

## Supporting information

S1 Checklist (DOC)

S1 File Appendix study protocol–version EN-PT. (DOCX)
