# Supplementary material for: The effect of mindfulness on decision-making, inhibitory control, and impulsivity of substance use disorder in-treatment patients: A randomized clinical trial
Source: PLoS One. 2023 Nov 7;18(11):e0293502. doi: 10.1371/journal.pone.0293502 (PMC10629638; doi:10.1371/journal.pone.0293502)
Supplement: S1 File — (DOCX) [file pone.0293502.s002.docx]

# **Supporting Information**

Please upload a copy of your study protocol that was approved by your ethics committee/IRB as a Supporting Information file. By the study protocol, we mean the complete and detailed plan for the conduct and analysis of the trial approved by the ethics committee/IRB. Please send this in the original language. If this is in a language other than English, please also provide a translation.

**Study Protocol**

**Translated Version (English)**

**GOAL**

**Main goal**

To investigate and compare the effects of the 8-week MBRP protocol (Experimental Group, GE) and relaxation practices (Control Group, GC) on different aspects of impulsivity among people with Alcohol Use Disorders undergoing treatment in a Therapeutic Community *(Please be aware that the project has been amended by #2.782.832 and # 3.557.636 CEP including other substances and comparing to Treatment as Usual instead of relaxation practices group due to observances on Pilot trial).*

**Specific goals**

Evaluate the performance of the EG participants, compared to the GC in relation to the following parameters:

- Consequences Sensitivity and Decision Making evaluated by the Iowa Gambling Task;

- Subjective assessment of Impulsivity assessed by the UPPS-P (Urgency, Premeditation, Perseverance and Sensation Seeking – Impulsive Behavior Scale – Short Version);

- Response inhibition evaluated by the Stroop Test;

- Changes in the Implicit Associations of Valence and Motivation evaluated by the different versions of the IAT (Alcohol - Implicit Association Test);

- Subjective assessment of Executive Functions evaluated by the BRIEF-A (Behavior Rating Inventory of Executive Function – Adult Version).

**METHOD**

The present study is one of the projects that form part of a randomized controlled clinical trial entitled “Alcohol dependence: a longitudinal study of Mindfulness-Based Relapse Prevention (MBRP)”. Therefore, this project will be carried out concurrently with other scientific initiation (1) and doctoral (1) research projects. It consists of a longitudinal study with data collected through the application of questionnaires and computerized tasks that assess cognition in patients hospitalized for the treatment of SUT.

**Participants**

Participants will be recruited in a Therapeutic Community called Instituto Padre Haroldo located at Rua Doutor João Quirino do Nascimento, 1601, Jardim Boa Esperança – Campinas/SP. Later, other partnerships will be established to increase the sample as well as to increase the variability of the setting.

The sample should consist of 192 people, considering the sample per group of 30 participants.

1) both genders,

2) who are literate;

3) may or may not have the diagnosis of AUT according to psychiatric evaluation;

4) who have been in treatment at the partner institution for at least 15 days;

5) who have been abstinent for the last 15 days, except for tobacco and caffeine;

6) who accept and are interested in participating in the research.

The exclusion criteria are:

1) have a medical history of serious neurological or psychiatric illnesses that could compromise their physical integrity or practice performance (Bipolar Affective Disorder, Dementia, Korsakoff Syndrome, among others);

2) are using psychoactive substances (report) - except tobacco and caffeine;

3) exhibit suicidal ideation or other risks to self or others

4) Psychotic Disorders.

The recruitment of participants will be carried out in the Therapeutic Community (described in the Procedures section) through an introductory lecture to clarify the interventions that will be offered and the tests that will be carried out. all participants

**STUDY DESIGN**

The study is a clinical, randomized, controlled trial.

**GROUPS**

Participants will be randomized into two groups: 8-week MBRP group in addition to usual care (Experimental Group, EG) and usual care group (Control Group, CG).

The Treatment As Usual offered by the therapeutic community consists of relapse prevention, group psychotherapy and 12 steps during the 8 weeks parallel to the EG.

The EG, in addition to the usual treatment offered by the institution, will receive the MBRP structured in eight weekly meetings lasting about two hours each, aiming to work on raising awareness of triggers, habitual patterns and automatic reactions that permeate the addictive process (BOWEN , SARAH; CHAWLA, NEHARIKA; COLLINS, SUSAM; WITKIEWITZ, 2009) composed of up to 20 people. During these meetings, four main meditation techniques are worked on: mindfulness of breathing, body scanning, meditative walking, and mindfulness movements. The specific themes of each session are: 1 – autopilot and relapse; 2 – conscience, triggers and craving; 3 – Mindfulness in daily life; 4 – Mindfulness in high-risk situations; 5 – acceptance and development of skills; 6 – seeing thoughts as thoughts; 7 – self-care and a balanced lifestyle; 8 – social support and continued practice.

The groups will be led by a facilitator chosen from within the research team who has consolidated meditative practice and is qualified in the aforementioned program, having been trained in national reference centers by the leader of the team that created the intervention protocol, Dr. Sarah Bowen.

Those from the CG who want to carry out the eight-week protocol offered to the EG will be offered a voucher to participate in the groups after the research in our outpatient unit

**INSTRUMENTS**

The instruments will be applied at two different times: before the first MBRP session (T0) and at the end of the MBRP protocol, equivalent to the 9th week of the MBRP (T1). For the control group will also be performed before and after eight weeks.

- Sociodemographic and clinical data questionnaire:

Self-completed questionnaire on gender, date of birth and age, profession, education, marital status, family income, who they live with, use of medications, date of entry into the institution and treatment phase. The diagnosis or presence of psychiatric disorders will be informed by the institution's psychiatrist (see ANNEX 1).

- Alcoholic and Non-Alcoholic Beverage Preference Questionnaire:

Questionnaire in which the interns must fill in the preference for five alcoholic beverages and five non-alcoholic beverages in order to standardize the preferred beverages for the culture and add them to the IAT test of motivation and IAT of adjectives (see ANNEX 2).

- WAIS-III Vocabulary Subtest and Cubes Subtest:

The Vocabulary and Cubes subtests are two subtests of the Wechsler Intelligence Scale for adults that can be used to assess estimated intellectual capacity. Patients who score equal to or greater than the median will be considered for the performance of the other tests, therefore, it will be used as a screening of patients (COUTINHO; NASCIMENTO, 2010).

- Five Facets of Mindfulness (FFMQ-Short Version):

FFMQ is a questionnaire translated, validated and adapted for Brazil (BARROS, 2014) that measures the mindfulness trait in five facets: observe, describe, non-reactivity, act with awareness and non-judgment of internal experience. It has 20 questions on a 5-point Likert scale (where 1 means “never or rarely true” and 5 “almost always or always true”) (see ANNEX 3).

- Urgency, Premeditation, Perseverance and Sensation Seeking Impulsive Behavior Scale – Short Version (UPPSP - Short):

UPPSP is a scale developed by Cyders (2007) that contains the five facets of impulsivity: positive urgency, negative urgency, premeditation, perseverance and sensation seeking. It presents 20 items with answers on the Likert scale, being able to respond from 1 to 4 (from 1 “strongly agree” and 4 “strongly disagree”. Its Portuguese version was validated in Brazil by Sediyama (2017). The greater the impulsivity, the greater the individual scores in each category Application time varies, but can be up to 10 minutes (see ANNEX 4).

- Penn Alcohol Craving Scale (Penn):

PACS is a scale developed by Flanery (1999) with five items that measures self-report on issues that include: frequency, intensity and duration of cravings, ability to resist drinking. Provides an overall average of alcohol cravings over the past week. Each of the questions can be evaluated from 0 to 6. The filling time varies, but on average it is 5 minutes (see ANNEX 5).

- Stroop Test (ST):

Task that assesses inhibitory control ability, that is, the ability to modify one's perception and adapt them to the demands of the environment or to suppress the usual response in favor of a new demand. It was developed by Golden in 1978. The version used has three cards and the subject is instructed to read the name of the stimuli as quickly as possible. Each of the cards has an orientation, the first: the participant must read from left to right the name of each color described on the paper, second: he must read the name of the colors that are written and in the last step he will need to say the name of the color that is printed, and not the name of the written colors (SPREEN, O.; STRAUSS, 1998).

- Behavior Rating Inventory of Executive Function – Adult Version (BRIEF-A):

Self-report questionnaire that can be completed by persons aged 18-90 that assesses executive functions in adults and self-regulation in the environment. It comprises 75 items comprising different aspects of executive functions: inhibition, self-monitoring, organizing/planning, response initiation, task monitoring, emotional control, organizing, working memory, and change. The Brazilian version is in the process of being translated, adapted and validated.

- Computerized Task: Alcohol - Implicit Association Test – Valencia (Alcohol-IAT):

Computerized task used to evaluate implicit and automatic association on alcohol used by Ostafin (2013). The IAT will be presented on a computer by Inquisit Software (OSTAFIN; KASSMAN; WESSEL, 2013). The task consists of categorization of stimulus participants into four categories – two target categories (alcoholic beverage or non-alcoholic beverage) and two attribute categories (pleasant, good, happy, like, pleasant) or negative (horrible, bad, dislike, unhappy). , unpleasant). The beverage stimuli will be defined after completing the “Alcoholic and Non-Alcoholic Beverages Preference Questionnaire” in order to standardize for a specific population.

During the combination of blocks, the following will be presented: a) 20 trials of alcoholic beverages versus non-alcoholic beverages; b) 20 positive versus negative trials; c) 40 trials of alcoholic beverages + positive adjective versus non-alcoholic beverages; d) 40 trials same pairing as c); e) 20 trials with reversed attribute categories; f) 40 trials with the same pairing as f. A red X appears if the participant makes a mistake. Results are evaluated so that higher scores for alcohol-positive drinks indicate preference. Application time is 5 minutes. The IAT is calculated automatically through an algorithm called D (GREENWALD; NOSEK; BANAJI, 2003).

- Computerized Task: Implicit Association Test - Motivation (IAT-Alcohol):

Computerized task used to evaluate implicit and automatic association on alcohol used by Ostafin (2012). The IAT will be presented on a computer by Inquisit Software (OSTAFIN; BAUER; MYXTER, 2012). The task consists of categorization of stimuli participants into four categories – two target categories (alcoholic beverage or non-alcoholic beverage) and two categories of attributes related to motivation: Approach (advance, approach, next, forward, direction); Avoid (avoid, move away, escape, leave, withdraw). Stronger associations indicate greater affection-stimulus, will be answered more quickly since the implicit association is more consolidated. The beverage stimuli will be defined after completing the “Questionnaire on Preference for Alcoholic and Non-Alcoholic Beverages” in order to standardize for a specific population, but in this case, representative photos of the beverage will be included in the test.

- Computerized Task: Iowa Gambling Task (IGT):

Computerized task used to assess decision-making ability under uncertain conditions of reward and punishment. The electronic version of the IGT (BECHARA et al., 2001; BECHARA, 2005) is the most widely used and it is a game in which the participant receives feedback (punishment and reward) on the deck he has chosen. Allows providing numerical feedback on rewards and punishments, visual feedback on losses and gains in each play (a happy “emoticon” is shown when the balance is positive and a sad “emoticon” when the balance is negative) and the balance update at each move is also made available above the

The Brazilian version of the test was translated, constructed and validated (SCHNEIDER; PARENTE, 2006). Over 100 plays, and throughout them, the participant must choose among the four decks, two more advantageous and two disadvantageous, with the aim of accumulating as much money as possible. Each deck has 40 cards and clicking on it, the participant receives numerical feedback on how much he won and if he lost something. Deck “A” and “B” have earnings of R$100.00 to R$250.00 and deck “C” and “D” have earnings of R$50.00. The final profit varies from participant to participant, but what matters is the punishment, because in decks that pay more (A and B) are more disadvantageous, the punishment is greater than in decks that pay less (C and D). The monetary gain in decks A and B is much greater and short-term, but the losses are more intense in just one move, however, in decks E and D the loss occurs more recurrently and less intensely, despite the gains being of long term.

The score is based on the “total calculation”, which corresponds to the sum of the choices from the advantageous decks (C+D) minus the sum of the decks considered risky (A+B). The classification of the decision-making performance of the participants in adaptive, borderline or impaired is given according to the previously adopted cutoff points (BECHARA, 2005). In the case of scores greater than 18, decisional performance is classified as unimpaired; between -18 and 18, borderline; and below -18, impaired. The second score indicates how much learning took place during the task and is called “block calculation”, allowing the analysis of a learning curve. Application time is 5 minutes.

**PROCEDURES**

Participants will be undergoing treatment at the Therapeutic Community (CT) Instituto Padre Haroldo located at: Rua Doutor João Quirino do Nascimento, 1601, Jardim Boa Esperança – Campinas/SP in a closed regime. Initially, we will hold a lecture composed of all the interns in order to explain about the research that will be conducted at the TC and invite them to participate.

Then, those who applied to be research participants will undergo a screening that will be evaluated if they meet the inclusion criteria, if they meet the pre-established requirements, they will be invited to participate in the research and will fill out the TCLE. Enrolled participants will complete baseline (T0) tests prior to the intervention. The tests are: Sociodemographic and Clinical Questionnaire, Questionnaire on Preference for Alcoholic and Non-Alcoholic Beverages; FFMQ; UPPSP; PANAS and Penn Scale. Subsequently, we will carry out the tests in which the participants will have to fill in the Stroop Test and the BRIEF-A with paper and pen and via computer the tests: motivational IAT, valence IAT, IGT and BRIEF-A.

After completing these tests, participants will be randomized between groups: EG and GC. The EG intervention is MBRP added to usual care, and the GC receives usual care. This process will take 8 weeks for both groups and all participants will complete a support material withdrawal monitoring with a collaborator in the therapeutic community itself.

After the intervention, there will be the post-intervention (T1) in which the participants will fill in the questionnaires previously completed during Baseline and T0, namely: FFMQ, UPPS-P, PANAS, Penn Scale, Stroop Test, motivational IAT, IAT valence, IGT and BRIEF-A. Below, a flowchart will briefly explain the steps described in the procedures section:


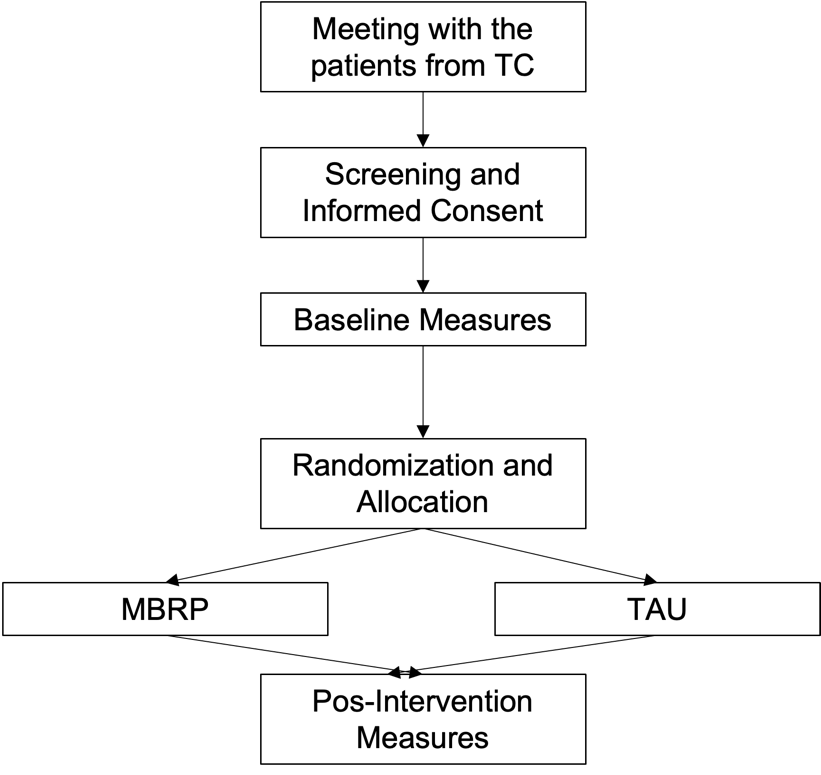


**Study Protocol**

**Original Version (Portuguese - BR)**

# **OBJETIVO**

## **Objetivo Geral**

Investigar e comparar os efeitos do protocolo de 8 semanas de MBRP (Grupo Experimental, GE), e a práticas de relaxamento (Grupo Controle, GC), em diferentes aspectos da impulsividade entre pessoas por Transtornos por Uso de Álcool em tratamento em uma Comunidade Terapêutica.

## **Objetivos Específicos**

Avaliar o desempenho dos participantes do GE, por comparação ao GC em relação aos seguintes parâmetros:

- Sensibilidade a consequências e Tomada de Decisão avaliado pelo Iowa Gambling Task;

- Avaliação subjetiva de Impulsividade avaliada pela UPPS-P (Urgency, Premeditation, Perseverance and Sensation Seeking – Impulsive Behavior Scale – Short Version);

- Inibição de resposta avaliado pelo Stroop Test;

- Alterações nas Associações Implícitas de Valência e de Motivação avaliado pelas diferentes versões do IAT (Alcohol - Implicit Association Test);

- Avaliação subjetiva das Funções Executivas avaliada pela BRIEF-A (Behavior Rating Inventory of Executive Function – Adult Version).

# **MÉTODO**

O presente estudo é um dos projetos integrantes de um ensaio clínico controlado e randomizado intitulado “Dependência do álcool: estudo longitudinal de Prevenção das Recaídas Baseadas em Mindfulness (MBRP)”. Portanto, este projeto será realizado concomitante à outros projetos de pesquisa de iniciação científica (1) e doutorado (1). Consiste em um estudo longitudinal com dados colhidos através da aplicação de questionários e tarefas computadorizadas que avaliam a cognição à pacientes internados para tratamento de TUS.

## **Participantes**

Os participantes serão recrutados em Comunidade Terapêutica denominada Instituto Padre Haroldo localizada no endereço: Rua Doutor João Quirino do Nascimento, 1601, Jardim Boa Esperança – Campinas/SP. Posteriormente, outras parcerias serão estabelecidas para o aumento da amostra bem como para aumentar a variabilidade do setting.

A amostra deverá ser composta por 192 pessoas, tendo em vista amostra por grupo de 30 participantes.

1) ambos os sexos;

2) que sejam alfabetizados;

3) **podem ou não ter o diagnóstico de TUA de acordo com avaliação psiquiátrica;**

4) **que estejam em tratamento na instituição parceira há pelo menos 15 dias internados;**

5) que estejam em abstinência nos últimos 15 dias, exceto **para tabaco cafeína;**

6) que aceitem e tenham interesse em participar da pesquisa.

Os critérios de exclusão são:

1. apresentarem histórico médico de doenças neurológicas ou psiquiátricas graves que possam comprometer a própria integridade física ou o desempenho da prática (Transtorno Afetivo Bipolar, Demências, Síndrome de Korsakoff, entre outros);
2. estiverem fazendo uso de substâncias psicoativas (relato) - exceto tabaco e cafeína;
3. apresentar ideação suicida ou outros riscos para si mesmo ou outros
4. **Transtornos Psicóticos.**

O recrutamento dos participantes será realizado em Comunidade Terapêutica (descrita na sessão *Procedimentos*) por meio de palestra introdutória de esclarecimento sobre as intervenções que serão ofertadas e os testes que serão realizados. Todos os participantes deverão fornecer consentimento para participar da pesquisa por meio do Termo de Consentimento Livre e Esclarecido (TCLE).

**Tipo de Estudo**

O estudo é um ensaio clínico, randomizado e controlado.

**Grupos**

Os participantes serão distribuídos de forma randomizada em dois grupos: grupo de 8 semanas do MBRP complementar ao tratamento usual (Grupo Experimental, GE) e grupo tratamento usual (Grupo Controle, GC).

O Tratamento Usual oferecido pela comunidade terapêutica consiste em prevenção de recaída, psicoterapia em grupo e 12 passos durante as 8 semanas paralelas ao GE.

O GE, além do tratamento usual oferecido pela instituição receberá o MBRP estruturado em oito encontros semanais com duração de cerca de duas horas cada, visando trabalhar a tomada de consciência dos gatilhos, dos padrões habituais e das reações automáticas que permeiam o processo aditivo (BOWEN, SARAH; CHAWLA, NEHARIKA; COLLINS, SUSAM; WITKIEWITZ, 2009) composto por até 20 pessoas. Durante esses encontros, trabalha-se com quatro técnicas principais de meditação: atenção plena (Mindfulness) na respiração, escaneamento corporal, caminhada meditativa e movimentos com atenção plena. Os temas específicos de cada sessão são: 1 – piloto automático e recaída; 2 – consciência, gatilhos e fissura; 3 – Mindfulness na vida diária; 4 – Mindfulness em situações de alto risco; 5 – aceitação e desenvolvimento de habilidades; 6 – enxergando os pensamentos como pensamentos; 7 – autocuidado e estilo de vida equilibrado; 8 – suporte social e prática continuada.

Os grupos serão conduzidos por um facilitador escolhido dentro da equipe de pesquisa que possua prática meditativa consolidada e com habilitação no referido programa, tendo sido treinado em centros de referência nacional pela líder da equipe idealizadora do protocolo, Dra. Sarah Bowen.

Para aqueles do GC que quiserem realizar o protocolo de oito semanas oferecido para o GE será ofertado um voucher para participar dos grupos após a pesquisa em nossa unidade ambulatorial

## **Instrumentos**

Os instrumentos serão aplicados em dois momentos diferentes: anterior à primeira sessão de MBRP (T0) e ao final do protocolo de MBRP, equivalente à 9° semana do MBRP (T1). Para o grupo controle também serão realizados antes e após oito semanas.

- **Questionário de dados sociodemográficos e clínico:**

Questionário de autopreenchimento sobre gênero, data de nascimento e idade, profissão, escolaridade, estado civil, renda familiar, com quem mora, uso de medicações, data de entrada na instituição e fase de tratamento. O diagnóstico ou presença de transtornos psiquiátricos será informado pelo psiquiatra da instituição (veja ANEXO 1).

- **Questionário de Preferência de Bebidas Alcóolicas e Não-Alcóolicas:**

Questionário em que os internos deverão preencher a preferência sobre cinco bebidas alcóolicas e cinco bebidas não alcóolicas com objetivo de padronizar para a cultura as bebidas de preferência e adicioná-las ao teste IAT de motivação e IAT de adjetivos (veja ANEXO 2).

- **Substeste Vocabulário e Subteste Cubos do WAIS-III:**

Os subtestes Vocabulário e o Cubos tratam-se de dois subtestes da Escala de Inteligência Wechsler para adultos que podem ser utilizados para avaliar a capacidade intelectual estimada. Será considerado pacientes que pontuarem igual ou maior que mediano para a realização dos demais testes, portanto, será utilizado como uma triagem dos pacientes (COUTINHO; NASCIMENTO, 2010).

- **Five Facets of Mindfulness (FFMQ-Short Version):**

FFMQ é um questionário traduzido, validado e adaptado para o Brasil (BARROS, 2014) que mede o traço de mindfulness em cinco facetas: observar, descrever, não reatividade, agir com consciência e não julgamento da experiência interna. Possui 20 questões de escala Likert de 5 pontos (sendo que 1 significa “nunca ou raramente verdadeiro” e o 5 “quase sempre ou sempre verdadeiro”) (veja ANEXO 3).

- **Urgency, Premeditation, Perseverance and Sensation Seeking Impulsive Behavior Scale – Short Version (UPPSP - Short):**

UPPSP é uma escala desenvolvida por Cyders (2007) que contém as cinco facetas impulsividade: urgência positiva, urgência negativa, premeditação, perseverança e busca por sensações. Apresenta 20 itens com respostas na escala Likert, podendo responder de 1 à 4 (de 1 “concorda fortemente” e 4 “discorda fortemente”. Sua versão em português foi validada no Brasil por Sediyama (2017). Quanto maior impulsividade, maior o indivíduo pontua em cada categoria. O tempo de aplicação varia, mas pode ser até 10 minutos (veja ANEXO 4).

- **Penn Alcohol Craving Scale (Penn):**

PACS é uma escala desenvolvida por Flanery (1999) com cinco itens que mede o auto relato sobre questões que incluem: frequência, intensidade e duração da fissura, habilidade de resistir a bebida. Fornece uma média geral sobre a fissura de álcool na última semana. Cada uma das questões pode ser avaliada de 0 à 6. O tempo de preenchimento varia, mas em média são 5 minutos (veja ANEXO 5).

- **Stroop Test (ST):**

Tarefa que avalia habilidade de controle inibitório, ou seja, habilidade de modificar sua percepção e adequá-las a demanda do ambiente ou suprimir a resposta habitual em favor de uma nova demanda. Foi desenvolvido por Golden em 1978. A versão utilizada possui três cartões e o sujeito é orientado a ler o mais rápido possível o nome dos estímulos. Cada um dos cartões possui uma orientação sendo que, o primeiro: o participante deve ler da esquerda para direita o nome de cada cor descrito no papel, segundo: deve ler o nome das cores que estão escritas e na última etapa ele precisará dizer o nome da cor que está impressa, e não o nome das cores escritas (SPREEN, O.; STRAUSS, 1998).

- **Behavior Rating Inventory of Executive Function – Adult Version (BRIEF-A):**

Questionário de autorelato que pode ser preenchido por pessoas de 18-90 anos de idade que avalia as funções executivas em adultos e autoregulação no ambiente. Compreende 75 itens que compreendem diferentes aspectos das funções executivas: inibição, automonitoramento, organização/planejamento, iniciação de resposta, monitoramento de tarefa, controle emocional, organização, memória de trabalho e mudança. A versão brasileira está em processo de tradução, adaptação e validação.

- **Tarefa Computadorizada: Alcohol - Implicit Association Test – Valencia (Alcohol-IAT):**

Tarefa computadorizada utilizada para avaliar associação implícita e automática sobre álcool utilizada por Ostafin (2013). O IAT será apresentado em um computador pelo Inquisit Software (OSTAFIN; KASSMAN; WESSEL, 2013). A tarefa consiste na categorização dos participantes dos estímulos em quatro categorias – duas categorias alvo (bebida alcóolica ou bebida não alcóolica) e duas categorias de atributos (agradável, bom, feliz, como, agradável) ou negativo (horrível, ruim, antipatia, infeliz, desagradável). Os estímulos de bebidas serão definido após preenchimento do “Questionário de Preferência de Bebidas Alcóolicas e Não-Alcóolicas” a fim de padronizar para população específica.

Durante a combinação dos blocos serão apresentados: a) 20 trials bebidas alcóolicas versus bebidas não alcóolicas; b) 20 trials positivo versus negativo; c) 40 trials de bebidas alcóolicas + adjetivo positivo versus bebidas não alcóolicas; d) 40 trials mesmo pareamento de c); e) 20 trials com categorias de atributos revertidos; f) 40 trials com o mesmo pareamento que f. Uma X vermelho aparece caso o participante cometa algum erro. Os resultados são avaliados de modo que escores mais altos para bebidas alcóolicas-positivas indicam preferência. O tempo de aplicação é 5 minutos. O IAT é calculado automaticamente através de um algoritmo chamado D (GREENWALD; NOSEK; BANAJI, 2003).

- **Tarefa Computadorizada: Implicit Association Test - Motivação (IAT-Alcohol):**

Tarefa computadorizada utilizada para avaliar associação implícita e automática sobre álcool utilizada por Ostafin (2012). O IAT será apresentado em um computador pelo Inquisit Software (OSTAFIN; BAUER; MYXTER, 2012). A tarefa consiste na categorização dos participantes dos estímulos em quatro categorias – duas categorias alvo (bebida alcóolica ou bebida não alcóolica) e duas categorias de atributos relacionadas motivação: Approach (avance, aproxime, próximo, em frente, direção); Avoid (evite, afaste, escape, saia, retire). Associações mais fortes indicam maior afeto-estímulo, serão respondidas mais rapidamente visto que a associação implícita é mais consolidada. Os estímulos de bebidas serão definidos após preenchimento do “Questionário de Preferência de Bebidas Alcóolicas e Não-Alcóolicas” a fim de padronizar para população específica, mas neste caso serão colocados no teste fotos representativas da bebida.

Durante a apresentação dos blocos serão apresentados: 20 trials bebidas alcóolicas versus bebidas não alcóolicas; 2) 20 trials approach e avoid; 3) 40 trials de bebidas alcóolicas + approach versus bebidas não alcóolicas + avoid; 4) 20 trials bebidas não alcoólicas + bebidas alcoólicas, 5) 40 trials de bebida não alcóolica / approach versus bebidas alcóolicas e categoria avoid. O IAT é calculado automaticamente através de um algoritmo chamado D (GREENWALD; NOSEK; BANAJI, 2003).

- **Tarefa Computadorizada: Iowa Gambling Task (IGT):**

Tarefa computadorizada utilizada para avaliar a capacidade de tomada de decisão em condições incertas de recompensa e punição. A versão eletrônica do IGT (BECHARA et al., 2001; BECHARA, 2005) é a mais amplamente utilizada e trata-se de um jogo em que o participante recebe feedbacks (punição e recompensa) sobre o baralho que escolheu. Permite disponibilizar *feedback* numérico sobre as recompensas e punições, *feedback* visual sobre perdas e ganhos em cada jogada (é mostrado um “emoticon” feliz quando o saldo é positivo e um “emoticon” triste quando o saldo é negativo) e a atualização do saldo a cada jogada também é disponibilizado acima do

A versão brasileira do teste foi traduzida, construída e validada (SCHNEIDER; PARENTE, 2006). Ao longo de 100 jogadas, e ao longo delas o participante deve escolher dentre os quatro baralhos, dois mais vantajosos e dois desvantajosos, com objetivo de acumular mais dinheiro possível. Cada baralho possui 40 cartas e a clicar o participante recebe o feedback numérico sobre quanto ganhou e se perdeu algo. O baralho “A” e “B” possui rendimento de R$100,00 à R$250,00 e o baralho “C” e “D” possuem ganhos de R$50,00. O lucro final varia de participante para participante, mas o que importa é a punição, pois nos baralhos que pagam mais (A e B) são mais desvantajosos a punição é maior do que em baralhos que pagam menos (C e D). O ganho monetário no baralho A e B é muito maior e de curto prazo, mas as perdas são mis intensas em apenas uma jogada, porém, no baralho E e D a perda ocorre de forma mais recorrente e menos intensa, apesar dos ganhos serem de longo prazo.

A pontuação é de “cálculo total” que corresponde a soma das escolhas dos baralhos vantajosos (C+D) menos a soma dos baralhos considerados de risco (A+B). A classificação do desempenho decisório dos participantes em adaptativo, limítrofe ou prejudicado se dá conforme os pontos de corte adotados previamente (BECHARA, 2005). No caso de escores superiores a 18, o desempenho decisional é classificado como não prejudicado; entre -18 e 18, limítrofe; e inferiores a -18, prejudicado. A segunda pontuação indica o quanto houve de aprendizagem ao longo da tarefa e é denominada “cálculo por blocos”, permitindo a análise de uma curva de aprendizagem. O tempo de aplicação é 5 minutos.

## **PROCEDIMENTOS**

Os participantes estarão em tratamento na Comunidade Terapêutica (CT) Instituto Padre Haroldo localizada no endereço: Rua Doutor João Quirino do Nascimento, 1601, Jardim Boa Esperança – Campinas/SP em regime fechado. Inicialmente realizaremos uma palestra composta por todos os internos com objetivo de explicar acerca das pesquisas que serão conduzidas na CT e convidá-los a participar.

Em seguida, àqueles que se candidataram em serem participantes de pesquisa passarão por uma triagem que será avaliado se preenchem os critérios de inclusão, caso atendam os requisitos pré-estabelecidos serão convidados à participar da pesquisa e preencherão o TCLE. Os participantes incluídos preencherão testes baseline (T0) antes da intervenção. Os testes são: Questionário Sociodemográfico e Clínico, Questionário sobre Preferência de Bebidas Alcóolicas e Não-Alcóolicas; FFMQ; UPPSP; PANAS e Penn Scale. Posteriormente, realizaremos os testes em que os participantes terão que preencher com papel e caneta o teste Stroop Test e o BRIEF-A e via computador os testes: IAT motivacional, IAT valência, IGT e BRIEF-A.

Após o preenchimento desses testes, os participantes serão randomizados entre os grupos: GE e GC. A intervenções GE é o MBRP adicionado ao tratamento usual e, o GC recebe o tratamento usual. Este processo levará 8 semanas para ambos os grupos e todos os participantes preencherão um monitoramento de retirada do material de apoio com colaborador na própria comunidade terapêutica.

Após a intervenção, haverá o pós-intervenção (T1) em que os participantes preencherão novamente os questionários previamente preenchidos durante a Baseline e o T0, sendo eles: FFMQ, UPPS-P, PANAS, Penn Scale, Stroop Test, IAT motivacional, IAT valência, IGT e BRIEF-A. A seguir, um fluxograma explanará resumidamente as etapas descritas na sessão *procedimento*s:


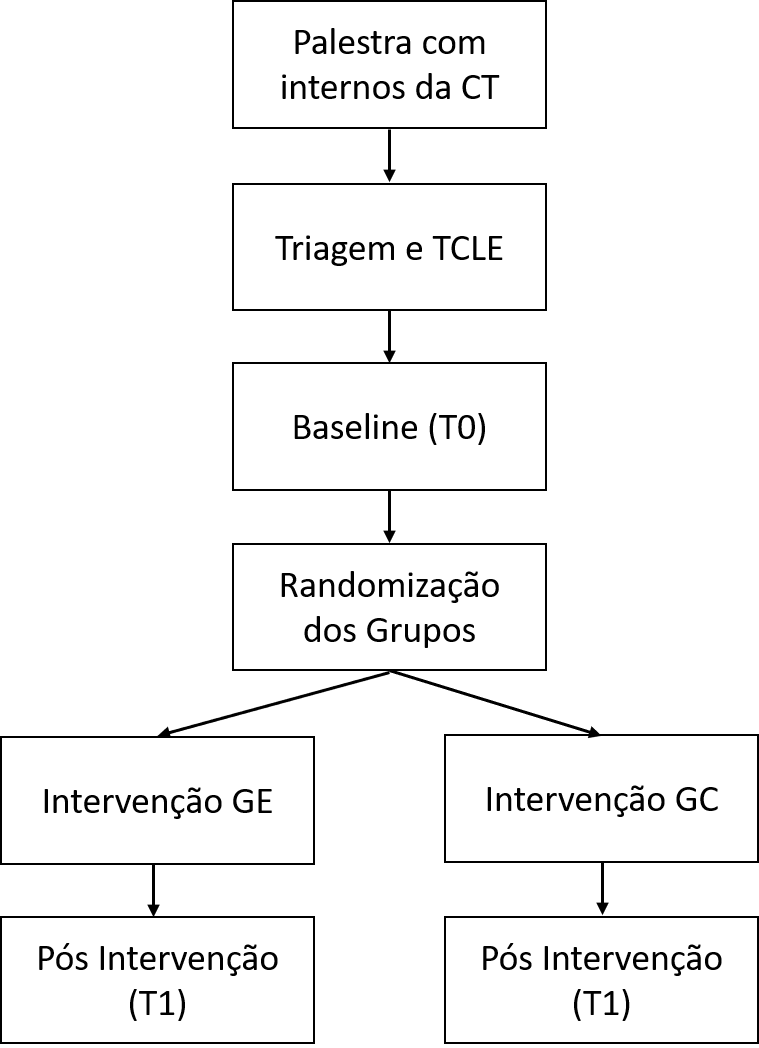


*Fluxograma com nome das principais etapas do procedimento realizado com os participantes.*
